# Acute doses of 3 and 6 mg/kg caffeine produce outcome- and phase-specific improvements in male 100-m sprinters: A randomized, double-blind, placebo-controlled crossover trial

**DOI:** 10.1371/journal.pone.0361864

**Published:** 2026-11-10

**Authors:** Tao Mei, Jianing Li, Xiangang Yang

**Affiliations:** 1 China Institute of Sport and Health Science, Beijing Sport University, Beijing, China; 2 Beijing Key Laboratory of Sports Performance and Skill Assessment, Beijing Sport University, Beijing, China; 3 Key Laboratory for Performance Training and Recovery of the General Administration of Sport of China, Beijing Sport University, Beijing, China; 4 Football College, Hebei Sport University, Shijiazhuang, China; University of Castilla-La Mancha, SPAIN

## Abstract

Evidence is limited on whether commonly used caffeine doses provide uniform benefits across sprint phases and performance outcomes in trained sprinters. We compared the acute effects of 3 and 6 mg/kg caffeine in a randomized, double-blind, placebo-controlled, three-condition crossover trial. Twenty-one male 100-m sprinters completed placebo, 3 mg/kg caffeine, and 6 mg/kg caffeine trials separated by at least 7 days. Sixty minutes after ingestion, participants completed two maximal 60-m sprints, followed by countermovement jump, squat jump, and 10-s Wingate tests. Sprint times, derived split times, start reaction time, starting-block rate of force development, stride variables, and perceived exertion were also assessed. Linear mixed-effects models adjusting for treatment period and randomized sequence showed clear treatment effects for cumulative 10-, 30-, and 60-m times (all p < 0.001). Relative to placebo, 3 mg/kg shortened these times by 0.056, 0.083, and 0.123 s, whereas 6 mg/kg shortened them by 0.075, 0.115, and 0.175 s. Compared with 3 mg/kg, 6 mg/kg further reduced 30- and 60-m times by 0.032 and 0.052 s, respectively, whereas the additional 0.019-s reduction at 10 m was not statistically significant in the primary mixed-effects model. Both doses improved reaction time, perceived exertion, jump height, Wingate peak and mean power, and starting-block rate of force development relative to placebo. Additional benefits of 6 mg/kg were evident for jump height and rate of force development, but not for reaction time, perceived exertion, or Wingate mean power; Wingate peak power did not improve further with the higher dose. Stride length and frequency were unchanged. These findings indicate outcome-specific dose responses, with 3 mg/kg improving multiple performance outcomes and 6 mg/kg providing additional benefits most consistently for vertical jump height, starting-block rate of force development, and cumulative performance over the first 60 m.

**Trial registration:**

Chinese Clinical Trial Registry, ChiCTR2600128594.

## Introduction

Caffeine is one of the most widely used acute ergogenic aids in competitive sport. It readily crosses the blood-brain barrier and acts primarily through antagonism of adenosine receptors, thereby attenuating inhibitory signaling in the central nervous system, increasing alertness and neural activation, and reducing pain and perceived exertion [1,2]. A substantial body of evidence shows that caffeine can enhance endurance, strength, and explosive performance, and current practical recommendations generally support ingestion of 3–6 mg/kg body mass approximately 60 min before exercise [1]. However, this recommended range does not imply that 3 and 6 mg/kg produce equivalent effects across all exercise tasks, nor that a higher dose necessarily yields a greater performance benefit. Classic dose-response research showed that plasma caffeine and catecholamine concentrations may continue to rise as caffeine dose increases, whereas improvements in exercise performance do not follow a simple linear pattern [3]. These circulating responses should not be interpreted as direct mechanical determinants of sprint performance. Their potential relevance is indirect: adenosine-receptor antagonism and systemic stimulation may increase central drive and motor-unit recruitment, but any sprint benefit must ultimately be expressed through task-specific mechanical changes such as faster force development, more effective horizontal force application, or altered support timing [1,4–6,14]. Thus, greater biochemical stimulation does not necessarily translate into proportionally greater sprint kinematics or faster sprint times.

The 100-m sprint provides a representative event-specific context in which to address this question. Although a 100-m race typically lasts only approximately 10–12 s, the athlete does not run at a constant velocity throughout. Instead, performance progresses through start reaction, block clearance, acceleration, attainment of maximal velocity, and the maintenance or decline of velocity [4,5]. The first 30–50 m after the start is primarily an acceleration phase, during which the sprinter must rapidly generate substantial horizontal propulsive force within progressively shorter ground-contact times. At higher running velocities, further acceleration or velocity maintenance depends on the direction, magnitude, and timing of ground-reaction-force application [4,6]. Thus, even when caffeine improves the final time, its effect may originate from distinctly different phases of the sprint.

Existing research on caffeine and sprint exercise does not adequately resolve this issue. Previous studies have largely recruited soccer, basketball, or rugby players and have typically used repeated-sprint protocols in which several-second sprints alternate with short recovery intervals to simulate the intermittent demands of team sports [7–9]. Such tasks are influenced by recovery capacity, tolerance to repeated efforts, and aerobic metabolic contribution, and therefore differ from the single, continuous, maximal sprint performed in a 100-m track event. Some studies have reported little effect of caffeine on repeated running or repeated Wingate performance, including no improvement in peak or mean power [7,8], whereas meta-analytic evidence indicates small but significant overall benefits for Wingate peak and mean power [10]. Training background may further condition acute responses because baseline neuromuscular capacity, fatigue resistance, and technical organization differ between team-sport athletes and highly trained sprinters. Accordingly, caffeine effects observed in repeated-sprint team-sport protocols should not be assumed to transfer directly to a single maximal sprint performed by specialist sprinters.

Evidence most closely resembling a single track sprint remains limited. Matsumura et al. [11] conducted a field-based 100-m trial using 6 mg/kg caffeine and found that adjusted overall performance improved, with most of the difference emerging within the first 60 m. This finding suggests that caffeine may exert a greater influence on the start and acceleration phases than on later velocity maintenance. However, that study included only one caffeine dose and therefore could not determine whether 3 mg/kg would provide a comparable benefit or whether increasing the dose to 6 mg/kg would yield additional improvement in specific sprint segments. Direct comparison of 3 and 6 mg/kg in trained sprinters is therefore useful for clarifying phase-specific and outcome-specific effects. Existing evidence has not established a consistent difference between these doses. On the one hand, 3 mg/kg can produce clear central stimulation and ergogenic effects in various exercise tasks, and increasing the dose to 6 mg/kg does not necessarily further improve endurance or short-duration sprint performance [3,9]. On the other hand, jumping, throwing, and resistance tasks that place greater demands on rapid motor-unit recruitment and short-duration force production have sometimes shown larger gains at higher doses [12,13]. These findings suggest that caffeine dose-response patterns may vary according to the exercise task and outcome assessed, and that any additional effect of a higher dose may be confined to specific components of performance. Comparing doses only by a single overall time may therefore obscure differences among sprint phases and related performance capacities.

Changes in sprint time should also be examined alongside responses that are closely related to event-specific performance. Short-distance sprinting depends not only on the final performance outcome but also on start reaction, rapid force generation, whole-body explosive capacity, technical organization, and perceived exertion. Caffeine has been shown to enhance neuromuscular excitability, explosive performance, and rate of force development (RFD). RFD reflects the rate at which force rises over a short time interval and is particularly relevant to the start and acceleration phases, in which force must be produced within limited support times [14,15]. However, most available evidence comes from jumping, resistance, or isometric tasks and cannot directly establish whether caffeine improves rapid horizontal force production from the starting blocks or whether changes in general performance capacities occur in parallel with event-specific sprint performance. Accordingly, split-time responses should be considered together with start reaction, rapid force production, overall explosive and anaerobic performance, kinematics, and perceived exertion to identify the phases in which each dose acts and the performance characteristics accompanying changes in sprint time.

In summary, it remains unclear whether 3 and 6 mg/kg caffeine exert comparable effects across different phases of sprinting in trained sprinters, and how changes in performance relate to start reaction, rapid force production, explosive performance, kinematics, and subjective responses. Therefore, this randomized, double-blind, placebo-controlled crossover study compared placebo, 3 mg/kg caffeine, and 6 mg/kg caffeine in male 100-m sprinters who had achieved the Chinese national second-class athlete standard. Cumulative and split times were used to evaluate performance over the first 60 m, together with vertical jump, Wingate power, start reaction time, RFD, step length, step frequency, and RPE. The aim was to determine the magnitude, principal phase of action, and associated performance responses of the two caffeine doses, thereby clarifying whether dose-related effects were consistent across the start and acceleration phases and related performance outcomes.

## Materials and methods

### Study design

This randomized, double-blind, placebo-controlled, three-condition crossover trial compared the acute effects of placebo (PLA; 0 mg/kg), 3 mg/kg caffeine (CAF3), and 6 mg/kg caffeine (CAF6) on event-specific performance in male 100-m sprinters. The study comprised four visits: one familiarization visit and three experimental trials. Participants were allocated by lot in blocks of three to one of three cyclic treatment sequences: A (PLA → CAF3 → CAF6), B (CAF3 → CAF6 → PLA), or C (CAF6 → PLA → CAF3), with seven participants assigned to each sequence. This allocation ensured that each treatment was represented by seven participants in each experimental period. Experimental trials were separated by at least 7 days. This interval was selected to minimize pharmacokinetic carryover and was substantially longer than the typical adult caffeine half-life of approximately 4–6 h (reported range, 1.5–10 h) [1]. Experimental control, supplementation timing, testing procedures, and the order of outcome assessments were identical across trials. Caffeine and placebo capsules were weighed, prepared, and coded by a researcher who was not directly involved in on-site testing. Participants and all staff responsible for capsule administration, testing, and data recording were unaware of capsule content. The participant flow and study design are shown in Fig 1.

**Fig 1:**
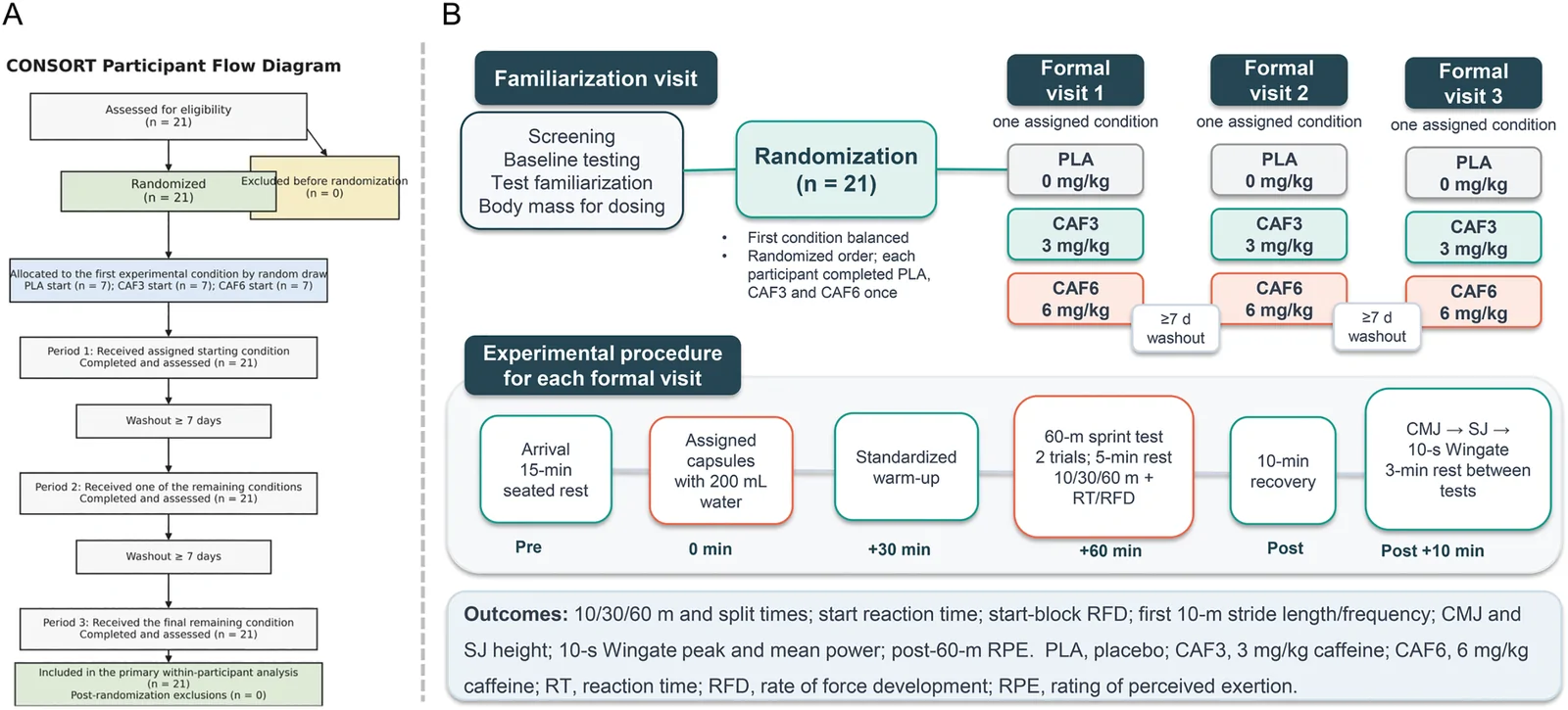
CONSORT participant flow diagram and study design. (A) Participant eligibility assessment, randomization, completion of the three experimental conditions, and inclusion in the primary within-participant analysis. (B) Experimental design. Each participant completed placebo (PLA), 3 mg/kg caffeine (CAF3), and 6 mg/kg caffeine (CAF6) conditions, separated by washout periods of at least 7 days. This trial is reported in accordance with CONSORT 2025 and the CONSORT extension for randomized crossover trials. The intervention is described in accordance with the TIDieR framework.

### Participants

Participants were male 100-m sprinters enrolled in the sport training program at Beijing Sport University. Inclusion criteria were: (1) 18–25 years of age; (2) performance meeting the Chinese national second-class standard for the 100 m during the study period; (3) at least 3 years of systematic sprint training; and (4) willingness to participate and ability to complete all testing. Exclusion criteria were: (1) known caffeine allergy or a history of marked adverse responses to caffeine ingestion; (2) an open or closed lower-limb joint injury within the previous 3 months; and (3) illness, pain, or other discomfort before testing that could impair maximal exercise. After screening, 21 participants completed all three experimental conditions.

No formal a priori power calculation was used to determine the target sample size. The target was feasibility-based and reflected the accessible pool of eligible male sprinters who met the national second-class performance standard during the recruitment window and could undertake the complete three-period crossover protocol. Twenty-one participants completed all three conditions. A sensitivity analysis based on the achieved sample size indicated that n = 21 provided 80% power to detect a standardized within-participant difference of d_*z*_ = 0.64 with a two-sided α = 0.05; using α = 0.0167 as a conservative threshold for three pairwise treatment comparisons, the corresponding detectable effect was d_*z*_ = 0.76. Smaller treatment differences may therefore have gone undetected.

Participants were recruited from 1 July 2026–5 July 2026. Prior to enrollment, all participants received written and verbal information regarding the study purpose, procedures, potential benefits, and risks, and provided written informed consent. The study was conducted in accordance with the Declaration of Helsinki and was approved by the Sports Science Experimental Ethics Committee of Beijing Sport University (approval no. 2026355H; approval date: 1 May 2026). The trial was retrospectively registered in the Chinese Clinical Trial Registry on 22 July 2026 (registration no. ChiCTR2600128594; https://www.chictr.org.cn/showproj.html?proj=333474). The delay in registration occurred because, at study initiation, the investigators did not recognize that this acute caffeine supplementation study in trained athletes met the applicable definition of an interventional clinical trial requiring prospective registration before participant enrollment. Once this requirement was recognized, the trial was retrospectively registered. Between the start of participant enrollment and trial registration, the intervention conditions, primary study procedures, primary outcomes, and randomization scheme were not modified on the basis of emerging study data or results. The authors confirm that all ongoing and related trials for this drug/intervention are registered. Participant characteristics are presented in Table 1, and participant flow is shown in Fig 1A.

**Table 1:** Participant characteristics. Values are mean ± standard deviation.

Age (years)	Height (cm)	Body mass (kg)	Personal best 100-m time (s)
20.0 ± 1.45	181.0 ± 4.87	74.0 ± 6.57	11.00 ± 0.24

### Pretrial controls

To reduce between-condition variation in time of day, diet, and preceding training load, the three experimental trials for each participant were scheduled at similar times of day. Exact session clock times, chronotype, habitual sleep-wake timing, recent sleep duration, and time since waking were not recorded, and testing was not scheduled relative to habitual wake time. Participants maintained their usual training program throughout the study but refrained from strenuous exercise and alcohol for 24 h before each trial and avoided sport-nutrition supplements other than those specified in the protocol. For 48 h before each trial, participants abstained from coffee, tea, energy drinks, cola, chocolate, and other caffeine-containing foods or beverages. They also refrained from eating for at least 2 h before arriving at the testing venue.

Before the first experimental trial, participants recorded the main foods and beverages consumed on the preceding day and on the day of testing. They were asked to replicate the same foods and meal timing as closely as possible before the subsequent two trials. Before each trial, investigators asked participants about their recent health status, caffeine intake, alcohol consumption, strenuous exercise, and supplement use, and habitual caffeine intake was recorded using a questionnaire. Compliance with all pretrial controls was verified verbally before each session. Testing equipment was inspected before each trial, and indoor temperature and relative humidity were recorded.

### Experimental procedures

The familiarization visit was used to explain and practice all testing procedures. On each experimental day, participants rested quietly for 15 min before ingesting the assigned capsules. In the caffeine conditions, commercially available Nutricost caffeine capsules (Nutricost, USA) were prepared to provide 3 or 6 mg/kg body mass. The placebo consisted of white flour enclosed in identical white capsules; the caffeine powder, flour, and capsules were all white in appearance. Participants swallowed all capsules with 200 mL of water. A standardized warm-up began 30 min after ingestion and consisted primarily of low-intensity jogging and dynamic stretching, sufficient to induce light perspiration without fatigue. An all-out 60-m sprint test was performed 60 min after ingestion. Each participant completed two trials separated by 5 min, and the faster trial was retained. After the 60-m test, participants rested seated for 10 min and then completed the countermovement jump (CMJ), squat jump (SJ), and 10-s Wingate test in a fixed order, with 3 min of rest between tests. The same order was used in all conditions.

### Outcome measures

#### 60-m Sprint times, start reaction time, and rate of force development.

Sprint testing was conducted on an indoor track. Participants used a crouch start from Kistler starting blocks with the corresponding starting system. A SmartSpeed portable timing system recorded cumulative times at 10, 30, and 60 m. Participants were instructed to sprint maximally through the 60-m finish immediately after the standardized start signal, without anticipating the signal or decelerating before the finish. Each participant completed two valid trials separated by 5 min, and the trial with the faster 60-m time was included in the main analysis. To characterize different acceleration phases, three split times were derived from the cumulative times: the 0–10-m split equaled the cumulative 10-m time; the 10–30-m split equaled the 30-m time minus the 10-m time; and the 30–60-m split equaled the 60-m time minus the 30-m time.

Start reaction time and starting-block force variables were acquired using the Kistler sprint-start system and processed in KiSprint software (version 4.1.0.12). During familiarization, participants adjusted the front and rear blocks to their customary distances and angles; these individualized settings were recorded operationally and reproduced before each experimental trial so that block geometry remained constant within each participant across conditions. Start reaction time was defined as the interval between presentation of the start stimulus and the system-detected onset of movement.

Rate of force development was calculated automatically from the force-time data by the system and exported for analysis. The prespecified performance outcomes were peak horizontal RFD (BW/s), front-block peak RFD (N·kg^−1^·s^−1^), and rear-block peak RFD (N·kg^−1^·s^−1^). Horizontal mean RFD (N·kg^−1^·s^−1^) was retained only for the exploratory change-score correlation analysis reported in S1 Table.

#### Step length and step frequency.

Kinematics during the initial acceleration phase were measured using an OptoJump optical measurement system. Ten transmitting and receiving units were positioned along both sides of the first 10 m of the track and synchronized with the Kistler starting system. Step length was defined as the horizontal distance between the toe of the support foot and the toe of the contralateral foot at the subsequent ground contact and was expressed in meters. Step frequency was calculated as the reciprocal of the step cycle, that is, 1/(ground-contact time + flight time), and was expressed in hertz. The database contained a representative mean step length and step frequency for the first 10 m under each condition for each participant.

#### Rating of perceived exertion.

Overall perceived exertion after sprinting was assessed using the Borg 6–20 rating of perceived exertion (RPE) scale. Scale anchors were explained using standardized instructions before testing, and RPE was obtained and recorded immediately after each 60-m sprint. A separate pre-sprint baseline RPE measure was not collected.

#### Countermovement jump and squat jump.

The CMJ and SJ were performed on a Kistler portable three-dimensional force platform. For the CMJ, participants rapidly descended from an upright position to approximately 90° of knee flexion and immediately jumped vertically with maximal effort. For the SJ, participants first assumed a squat position at approximately 90° of knee flexion and held it for 1–2 s before jumping vertically with maximal effort, without any visually detectable countermovement. Three valid trials were completed for each jump, and the greatest jump height calculated by the force-platform software was retained for analysis. Standardized instructions were provided before testing, and trials were repeated when clear technical errors occurred.

#### 10-s wingate power.

Anaerobic power was assessed using a 10-s Wingate test on a Monark 894E cycle ergometer. Saddle position was adjusted to each participant’s stature during familiarization, recorded, and reproduced during all three experimental trials. Resistance was set relative to body mass. On a standardized command, participants accelerated as rapidly as possible and cycled maximally for 10 s. Peak power (PP) and mean power (MP) over the 10-s test were recorded in watts. The load factor was 0.090 kg per kilogram of body mass.

### Statistical analysis

Continuous outcomes were analyzed using linear mixed-effects models to account for the three-period crossover design. Treatment (PLA, CAF3, and CAF6), experimental period (1, 2, and 3), and randomized sequence (A, B, and C) were included as fixed effects, with participant included as a random intercept to account for within-participant correlation. Models were estimated using restricted maximum likelihood. Fixed effects were evaluated using Wald chi-square tests. Pairwise treatment contrasts (CAF3 vs PLA, CAF6 vs PLA, and CAF6 vs CAF3) were derived from the fitted models and are reported as estimated mean differences with 95% confidence intervals. Holm adjustment was applied to the three pairwise comparisons for each outcome. Paired Hedges’ g_z_ was additionally calculated as a standardized descriptive effect size. Model residuals were assessed using histograms, Q–Q plots, skewness, kurtosis, and the Shapiro–Wilk test.

Gaussian generalized estimating equations (GEE) including the same treatment, period, and sequence terms were used as robustness analyses for continuous outcomes, with participant as the clustering variable, an exchangeable working correlation structure, and bias-reduced robust covariance estimates. RPE was analyzed as an ordinal outcome using a proportional-odds ordinal GEE with treatment, period, and sequence as predictors and participant-level clustering. Pairwise treatment effects for RPE are reported as odds ratios for a higher RPE category, with Holm-adjusted p values. Derived 0–10-, 10–30-, and 30–60-m split times were analyzed using the same mixed-effects framework as exploratory outcomes. Exploratory associations between placebo-referenced changes in 60-m time and corresponding changes in CMJ, SJ, Wingate peak and mean power, start reaction time, and RFD measures were examined using Spearman rank correlations, with p values adjusted using the Benjamini–Hochberg false-discovery-rate procedure.

The crossover design comprised three cyclic treatment sequences. Because the treatment received in the preceding period was structurally linked to the current treatment in periods 2 and 3, a first-order carryover effect could not be estimated independently from treatment, period, and sequence within the same model. Potential carryover was therefore assessed by examining period and sequence effects and by conducting a sensitivity analysis restricted to period 1, in which no participant had received a preceding study treatment. Experimental trials were separated by a washout interval of at least 7 days. All tests were two-sided, with statistical significance set at α = 0.05. All 21 randomized participants completed all three periods, yielding 63 participant-period observations; no outcome data were missing, and no imputation was performed. Analyses were conducted in Python 3 using pandas, SciPy, and statsmodels, and the analysis code is provided as S1 Code.

## Results

All 21 randomized participants completed all three experimental periods and all three treatment conditions, yielding 63 participant-period observations. No post-randomization losses, outcome exclusions, or missing observations occurred, and no imputation was required (Fig 1A). Across the continuous outcomes, no period effect reached statistical significance (all p ≥ 0.089) and no sequence effect reached statistical significance (all p ≥ 0.167). No systematic adverse-event data were collected.

A sensitivity analysis restricted to period 1 was conducted to examine treatment effects in the absence of a preceding study treatment. The CAF6-versus-PLA contrast was directionally consistent with the full crossover analysis for all continuous outcomes, and the CAF3-versus-PLA contrast was directionally consistent for 15 of 16 continuous outcomes; the only exception was stride frequency, which was also a null outcome in the primary analysis.

### Short-distance sprint performance

#### Cumulative 10-, 30-, and 60-m times.

Caffeine produced clear treatment effects on cumulative sprint performance [10 m: Wald χ^2^(2) = 47.552, p < 0.001; 30 m: χ^2^(2) = 79.203, p < 0.001; 60 m: χ^2^(2) = 90.065, p < 0.001]. Compared with PLA, CAF3 reduced 10-, 30-, and 60-m times by 0.056 s (95% CI: 0.033–0.078 s), 0.083 s (95% CI: 0.057–0.109 s), and 0.123 s (95% CI: 0.086–0.160 s), respectively. CAF6 reduced the respective times by 0.075 s (95% CI: 0.053–0.097 s), 0.115 s (95% CI: 0.089–0.141 s), and 0.175 s (95% CI: 0.138–0.212 s). All CAF3-versus-PLA and CAF6-versus-PLA comparisons remained significant after Holm adjustment (all p < 0.001), with large standardized effects for the cumulative sprint outcomes (|*g*_*z*_| = 0.896–1.664) (Figs 2 and 3).

**Fig 2:**
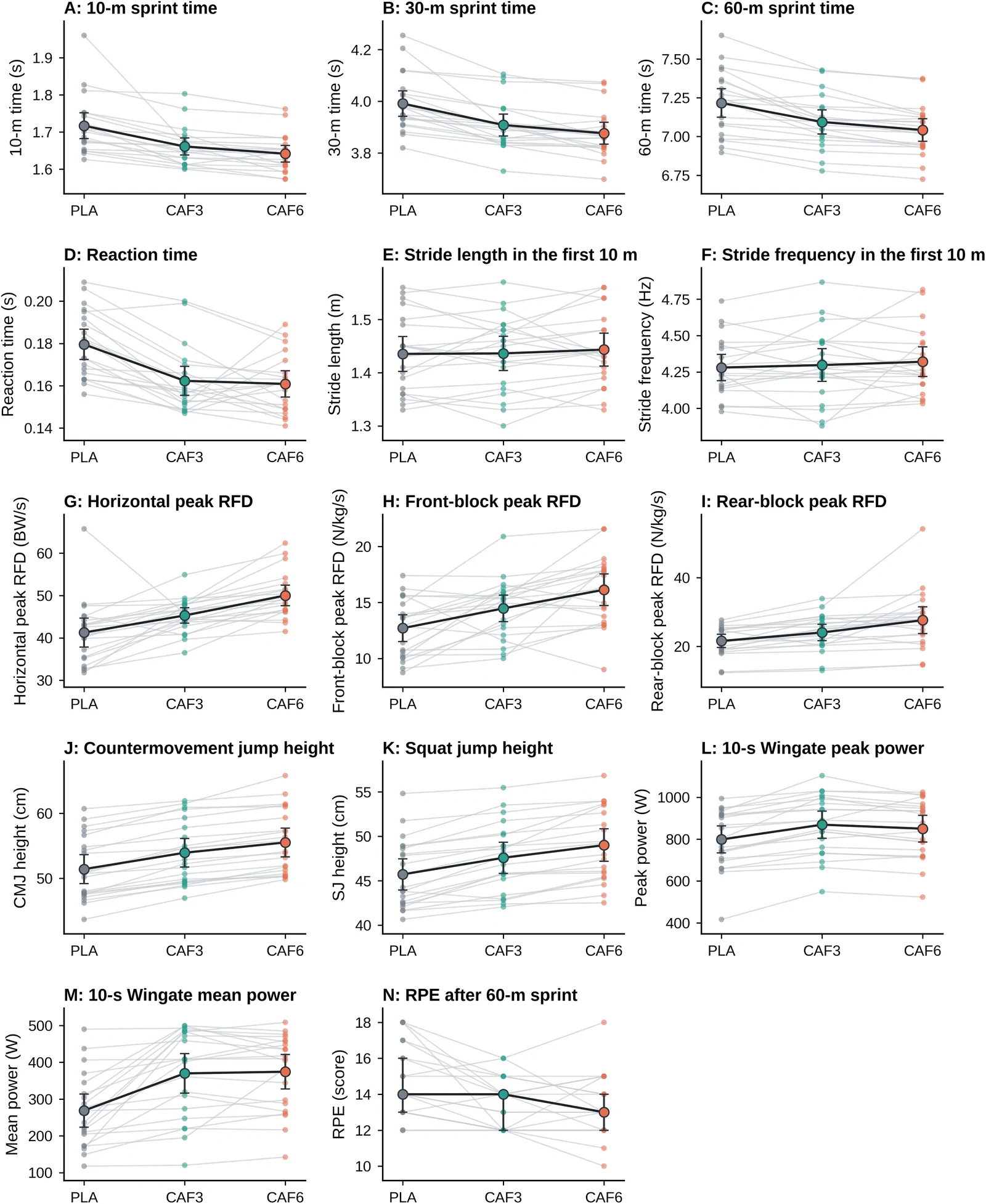
Individual trajectories and group-level estimates for the prespecified performance outcomes across placebo (PLA), 3 mg/kg caffeine (CAF3), and 6 mg/kg caffeine (CAF6). Thin gray lines connect repeated observations from individual participants, and small condition-colored points show individual values. Larger outlined condition-colored points, black connecting lines, and error bars represent group means and 95% confidence intervals for continuous outcomes; RPE is shown as median and interquartile range. Continuous-outcome inference was based on crossover-adjusted linear mixed-effects models. RPE inference used an ordinal generalized estimating equation model adjusted for period and sequence.

**Fig 3:**
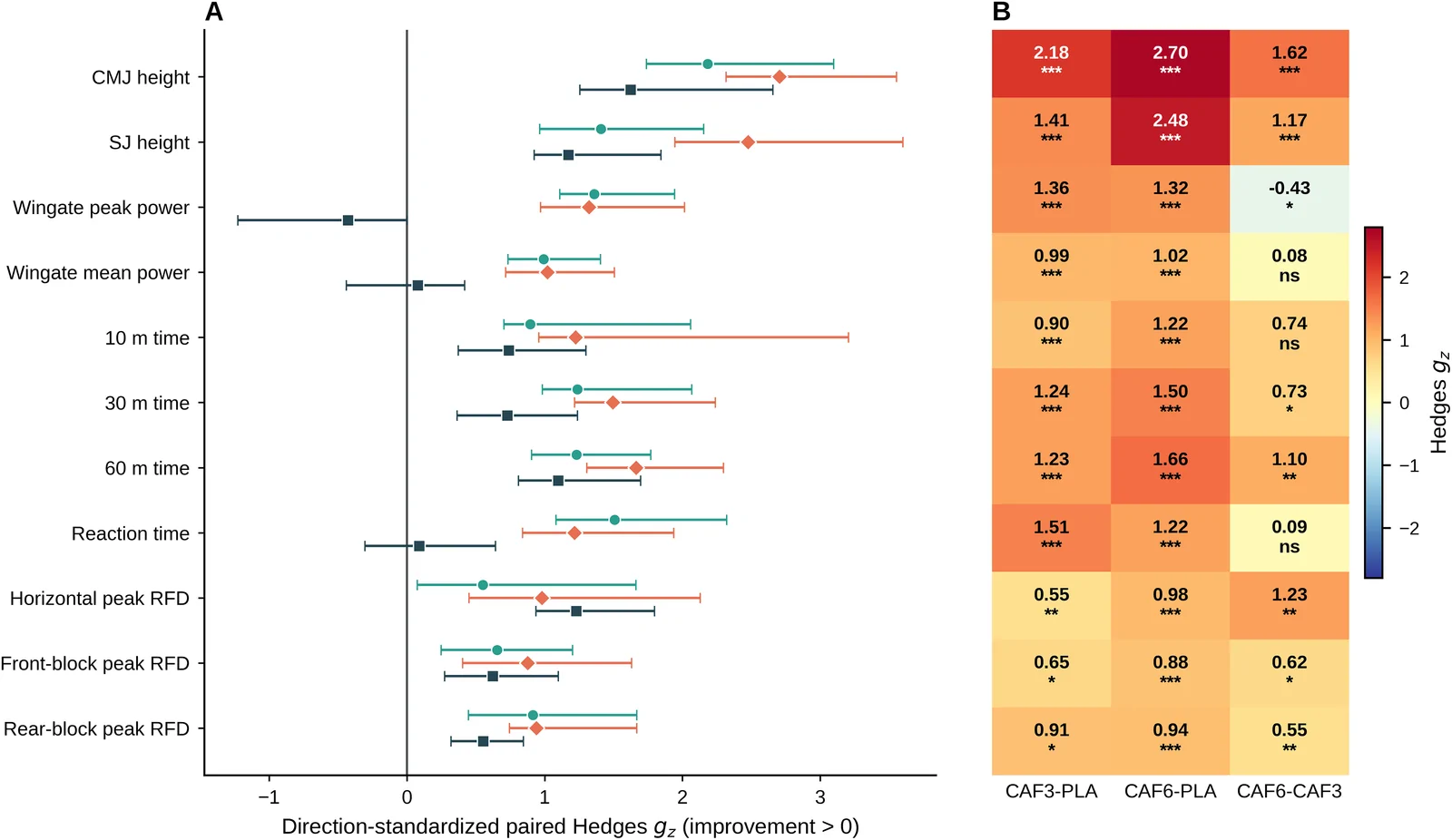
Pairwise standardized effects of 3 and 6 mg/kg caffeine on sprint-related performance outcomes. (A) Paired Hedges’ *g*_*z*_ estimates with participant-level bootstrap 95% confidence intervals; circles denote CAF3-PLA, diamonds denote CAF6-PLA, and squares denote CAF6-CAF3. (B) Heatmap of the corresponding standardized effect sizes. Sprint and reaction times were directionally inverted so that positive values indicate improvement. Significance symbols are based on Holm-adjusted treatment contrasts from the primary crossover-adjusted linear mixed-effects models. ns, nonsignificant; *p < 0.05, **p < 0.01, ***p < 0.001.

The higher dose produced additional cumulative sprint benefits at 30 and 60 m, but not conclusively at 10 m after crossover adjustment. Compared with CAF3, CAF6 reduced 10-m time by a further 0.019 s (95% CI: −0.003 to 0.042 s faster; pHolm = 0.085), 30-m time by 0.032 s (95% CI: 0.006–0.058 s faster; pHolm = 0.016), and 60-m time by 0.052 s (95% CI: 0.015–0.089 s faster; pHolm = 0.006). Thus, the numerical dose ordering was present at all three cumulative distances, but the CAF6-versus-CAF3 contrast at 10 m did not reach statistical significance in the primary mixed-effects model. At the individual level, 20 of 21 participants ran faster over 60 m under CAF3 than under PLA, and all 21 participants ran faster under CAF6 (Figs 2 and 3).

#### Exploratory analysis of split times.

Derived split times also showed treatment effects [0–10 m: Wald χ^2^(2) = 47.552, p < 0.001; 10–30 m: χ^2^(2) = 13.387, p = 0.001; 30–60 m: χ^2^(2) = 12.419, p = 0.002]. Over 0–10 m, CAF3 and CAF6 were 0.056 and 0.075 s faster than PLA, respectively; the additional 0.019-s reduction with CAF6 relative to CAF3 did not reach significance in the primary model (pHolm = 0.085). Over 10–30 m, CAF3 and CAF6 were 0.027 s (95% CI: 0.005–0.049 s, pHolm = 0.029) and 0.040 s (95% CI: 0.018–0.061 s, pHolm = 0.001) faster than PLA, whereas CAF6 did not differ from CAF3 (pHolm = 0.258). Over 30–60 m, CAF3 and CAF6 were 0.040 s (95% CI: 0.006–0.074 s, pHolm = 0.041) and 0.060 s (95% CI: 0.026–0.094 s, pHolm = 0.002) faster than PLA, respectively; the two caffeine doses did not differ significantly (pHolm = 0.252). The CAF3-versus-PLA contrast over 30–60 m was not significant in the robust GEE sensitivity analysis (pHolm = 0.108), whereas the CAF6-versus-PLA effect remained significant, so the lower-dose effect in this interval was interpreted cautiously (Fig 4).

**Fig 4:**
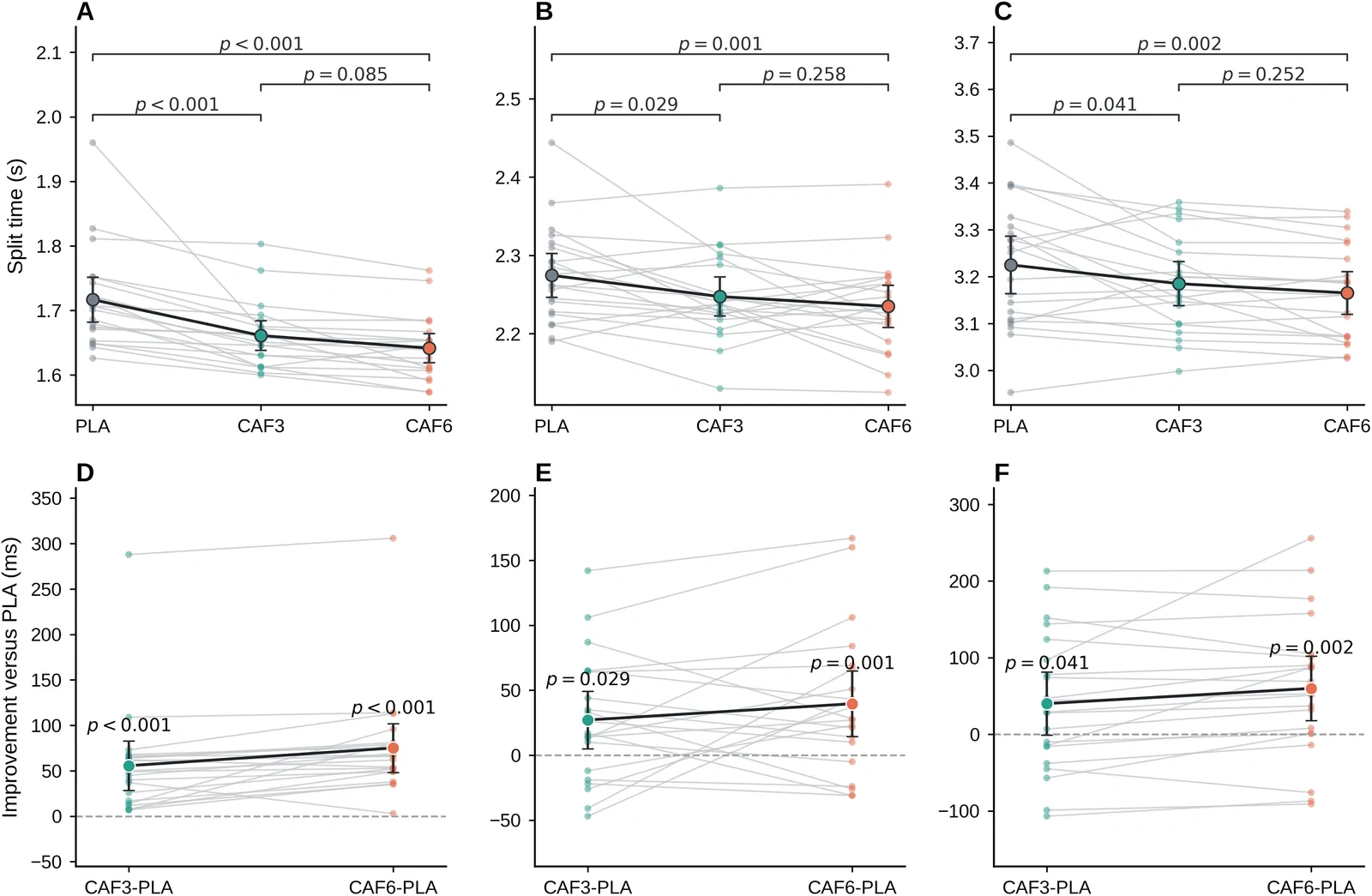
Individual trajectories for sprint split times and improvements relative to placebo. (A-C) Split times over 0-10, 10-30, and 30-60 m under PLA, CAF3, and CAF6. (D-F) Improvements under CAF3 and CAF6 relative to PLA, calculated as PLA split time minus caffeine-condition split time and expressed in milliseconds; positive values indicate shorter split times under caffeine. All 21 participants are displayed in every panel. Thin gray lines connect individual observations; larger condition-colored points, black connecting lines, and error bars represent group means and 95% confidence intervals. Displayed p values are Holm-adjusted treatment contrasts from the primary crossover-adjusted linear mixed-effects models; split-time analyses were exploratory.

#### Start reaction time and rate of force development.

Start reaction time showed a strong treatment effect [Wald χ^2^(2) = 49.153, p < 0.001]. Compared with PLA, CAF3 and CAF6 shortened reaction time by 17.2 ms (95% CI: 11.4–23.1 ms, pHolm < 0.001, *g*_*z*_ = −1.508) and 18.7 ms (95% CI: 12.9–24.5 ms, pHolm < 0.001, *g*_*z*_ = −1.217), respectively. The difference between the two caffeine doses was 1.5 ms (95% CI: −4.3 to 7.3 ms, pHolm = 0.619, *g*_*z*_ = −0.089), indicating no additional reaction-time benefit from CAF6.

All three prespecified starting-block RFD measures showed significant treatment effects [peak horizontal RFD: Wald χ^2^(2) = 34.285, p < 0.001; front-block peak RFD: χ^2^(2) = 25.892, p < 0.001; rear-block peak RFD: χ^2^(2) = 27.857, p < 0.001]. Compared with PLA, CAF3 significantly increased all three outcomes (Holm-adjusted p = 0.007–0.032), and CAF6 produced larger increases (all p < 0.001). CAF6 also exceeded CAF3 for peak horizontal RFD by 4.722 BW/s (95% CI: 1.791–7.652 BW/s, pHolm = 0.003), front-block peak RFD by 1.663 N·kg ⁻ ¹·s ⁻ ¹ (95% CI: 0.343–2.983, pHolm = 0.018), and rear-block peak RFD by 3.571 N·kg ⁻ ¹·s ⁻ ¹ (95% CI: 1.313–5.828, pHolm = 0.004) (Figs 2 and 3).

#### Step length and step frequency over the first 10 m.

Mean step length and step frequency over the first 10 m remained null findings after crossover adjustment. The treatment effect was nonsignificant for step length [Wald χ^2^(2) = 1.049, p = 0.592] and step frequency [χ^2^(2) = 1.617, p = 0.445]. All pairwise 95% confidence intervals crossed zero, all Holm-adjusted p values were ≥0.615, and |*g*_*z*_| was ≤ 0.327 (Figs 2 and 3).

#### Rating of perceived exertion.

RPE was 14 (13, 16), 14 (12, 14), and 13 (12, 14) under PLA, CAF3, and CAF6, respectively. The ordinal GEE showed a significant treatment effect [Wald χ^2^(2) = 16.860, p < 0.001] and a significant period effect [χ^2^(2) = 11.805, p = 0.003], whereas sequence was not significant (p = 0.465). Relative to PLA, the odds of reporting a higher RPE category were lower under CAF3 (OR = 0.236, 95% CI: 0.097–0.571, pHolm = 0.003) and CAF6 (OR = 0.176, 95% CI: 0.077–0.403, pHolm < 0.001). CAF6 did not differ significantly from CAF3 (OR = 0.746, 95% CI: 0.414–1.346, pHolm = 0.331) (Figs 2 and 3).

### Vertical jump height

Caffeine produced clear treatment effects on vertical jump performance [CMJ: Wald χ^2^(2) = 244.677, p < 0.001; SJ: χ^2^(2) = 141.423, p < 0.001]. Compared with PLA, CAF3 increased CMJ and SJ height by 4.99% and 4.14%, respectively, whereas CAF6 increased them by 8.12% and 7.24%. The standardized effects of both doses relative to PLA were large (Hedges’ *g*_*z*_ = 1.408–2.703). CAF6 increased CMJ height by 1.587 cm relative to CAF3 (95% CI: 1.066–2.107 cm, pHolm < 0.001, *g*_*z*_ = 1.624) and increased SJ height by 1.427 cm (95% CI: 0.883–1.971 cm, pHolm < 0.001, *g*_*z*_ = 1.171) (Figs 2 and 3).

### Anaerobic power during the 10-s wingate test

Both Wingate peak and mean power showed significant treatment effects [peak power: Wald χ^2^(2) = 60.693, p < 0.001; mean power: χ^2^(2) = 38.204, p < 0.001]. Compared with PLA, CAF3 and CAF6 increased peak power by 70.529 W (95% CI: 52.177–88.881 W, pHolm < 0.001, *g*_*z*_ = 1.360) and 51.389 W (95% CI: 33.037–69.741 W, pHolm < 0.001, *g*_*z*_ = 1.322), respectively. In the primary mixed-effects model, peak power under CAF6 was 19.140 W lower than under CAF3 (95% CI: 0.788–37.492 W lower, pHolm = 0.041, *g*_*z*_ = −0.427). However, this dose contrast was borderline and did not reach significance in the bias-reduced robust GEE sensitivity analysis (pHolm = 0.051); it was therefore interpreted as evidence that CAF6 did not provide an additional peak-power benefit rather than as a robust superiority of CAF3 (Figs 2 and 3).

For mean power, CAF3 and CAF6 produced increases of 101.195 W (95% CI: 63.302–139.089 W, pHolm < 0.001, *g*_*z*_ = 0.994) and 105.640 W (95% CI: 67.746–143.533 W, pHolm < 0.001, *g*_*z*_ = 1.020), respectively, relative to PLA. CAF6 differed from CAF3 by only 4.444 W (95% CI: −33.449 to 42.338 W, pHolm = 0.818, *g*_*z*_ = 0.079). Thus, the higher dose did not provide an additional benefit for Wingate mean power within the standardized post-sprint testing sequence.

Mixed-model residuals for Wingate peak power showed mild positive skewness (0.37), excess kurtosis (1.49), and some departure from normality (Shapiro-Wilk p = 0.022), whereas residuals for mean power were approximately symmetric (skewness = −0.08; excess kurtosis = −0.44; Shapiro-Wilk p = 0.521).

### Correlations between improvement in 60-m performance and biomechanical characteristics

Exploratory change-score correlations identified no association between improvement in 60-m performance and change in any individual candidate outcome that remained significant after FDR correction. Under CAF3 and CAF6, Spearman correlation coefficients between improvement in 60-m time and changes in CMJ, SJ, Wingate power, start reaction time, and the RFD measures ranged from −0.405 to 0.413, and all Benjamini-Hochberg FDR-adjusted q values were ≥0.565 (S1 Table).

## Discussion

The present study showed that the effects of caffeine on event-specific performance were not governed by a single dose threshold applicable to every outcome. Both doses improved vertical jump performance, 10-s Wingate power, cumulative 10-, 30-, and 60-m times, start reaction time, and starting-block RFD, while reducing perceived exertion after the 60-m sprint. After adjustment for period and randomized sequence, the additional benefits of 6 mg/kg over 3 mg/kg were most consistent for CMJ, SJ, cumulative 30- and 60-m times, and all RFD measures. The 10-m cumulative time was numerically lower under CAF6 than CAF3, but that contrast did not reach significance in the primary mixed-effects model. Wingate mean power, start reaction time, and RPE did not improve further beyond 3 mg/kg, and the higher dose did not provide a robust additional benefit for Wingate peak power. The split-time analyses likewise supported a caffeine effect across the acceleration phase, but direct separation of the two caffeine doses within the 10–30- and 30–60-m intervals was not evident. Thus, the dose response was outcome-specific rather than a uniform superiority of 6 mg/kg across acceleration performance.

### Dose-response characteristics of caffeine

The consistent dose ordering observed for vertical jump performance and RFD was one of the most notable findings. Compared with placebo, 3 mg/kg increased CMJ and SJ height by approximately 5.0% and 4.1%, respectively, whereas the corresponding improvements with 6 mg/kg reached approximately 8.1% and 7.2%. In a force-platform study using 3 mg/kg caffeine, Grgic observed not only greater jump height but also improvements in peak force, peak power, maximal rate of power development, and several velocity-related variables, with the most consistent responses occurring in velocity outcomes [17]. Muñoz et al. also reported that 3 mg/kg caffeine increased CMJ height by 4.2% and reduced 30-m sprint time by 1.6% in elite female handball players [18]. The magnitudes of the CAF3 effects on CMJ and 30-m time in the present study were similar, indicating that the benefits of a low caffeine dose for explosive actions and short-distance sprinting are not confined to a single sport.

The further improvements in CMJ and SJ under 6 mg/kg indicate that 3 mg/kg did not represent the upper dose threshold for vertical jump performance in these participants. The CMJ incorporates a prestretch and an eccentric-to-concentric transition, whereas the SJ begins from a relatively static position and relies more heavily on rapid concentric force production. The parallel dose-related increases in both jumps are consistent with an effect on performance capacities shared by the two tasks, although the present measurements do not identify a specific neural or muscular mechanism. The incremental-dose study by Pallarés et al. showed that the caffeine dose required for improvement depends on movement load and velocity demands: 3 mg/kg was sufficient for light-load, high-velocity actions, whereas higher doses were more beneficial for higher-load tasks [19]. The present findings are consistent with this task-dependent dose-threshold concept, while also indicating that dose-response patterns for vertical jumping in trained athletes cannot be inferred directly from general resistance-exercise tasks.

Starting-block RFD showed a dose-related response in an event-specific action. Peak horizontal, front-block, and rear-block RFD all followed the order 6 mg/kg > 3 mg/kg > placebo. Meta-analytic evidence indicates that 3–5 mg/kg caffeine has a small effect on RFD, whereas larger effects are observed with 6–10 mg/kg [15]. The present study extends this dose-related pattern from isometric and general strength tasks to horizontal force production from the starting blocks. Sprint starts require athletes to generate horizontal propulsive force rapidly within a very short block-contact period, making the early rate of force rise more task-relevant than maximal force alone [14]. The simultaneous increases in front- and rear-block RFD show that the response was not confined to one block. Nevertheless, a greater jump height does not imply that all mechanical power outcomes improve in parallel. Jump height is influenced by body mass, countermovement depth, push-off distance, and the force-velocity combination, and cannot be treated as a direct surrogate for maximal lower-limb power [20]. The study also did not include multi-joint isokinetic strength profiling; therefore, the observed changes in jump height should not be interpreted as a comprehensive assessment of joint-specific strength or neuromuscular capacity. In Grgic’s study, caffeine improved peak power and several velocity-related variables, whereas mean power, 300-ms impulse, and some mechanical variables were unchanged [17]. Research in elite volleyball players has likewise shown that caffeine does not affect all phase-specific CMJ variables uniformly [21].

In contrast to vertical jump performance and RFD, 10-s Wingate power plateaued after 3 mg/kg. Both CAF3 and CAF6 significantly increased peak and mean power, but the difference between doses was not consistent, and mean peak power was numerically highest under CAF3. This result does not negate the neuromuscular effects of higher-dose caffeine; rather, it indicates that these effects did not translate into further improvement in the cycling-power outcomes used in this study. The 10-s Wingate test emphasizes rapid attainment of high power, but its loading and technical demands differ from horizontal force production against the starting blocks. Consistent with Pallarés et al., a lower dose may be sufficient to improve high-velocity actions, whereas additional effects of a higher dose may be more likely in higher-load tasks [19]. The short-duration cycling results in the present study are compatible with this interpretation.

The order of testing also affects interpretation of the Wingate findings. Participants completed the CMJ, SJ, and Wingate test in sequence after the all-out 60-m sprint and 10 min of seated recovery. The Wingate test was therefore not an independent assessment performed in a fully recovered state, but rather a performance test conducted after a standardized preceding load. Jump and sprint performance are both sensitive to neuromuscular status, and different outcomes vary in their responsiveness to fatigue [22,23]. The identical test order across conditions ensured procedural comparability among treatments, but the faster 60-m performances under caffeine may have altered the magnitude of subsequent residual fatigue. No pre-sprint CMJ, SJ, or Wingate baseline was collected within each experimental session; consequently, the fatigue decrement produced by the preceding sprint cannot be quantified from the available data. The present study can therefore establish caffeine-related differences in these outcomes within the standardized post-sprint testing sequence, but it cannot isolate their pharmacological effects from preceding-sprint fatigue or determine whether the same dose-response pattern would occur in a fully recovered state.

Start reaction time and RPE showed a plateau similar to that observed for Wingate power. Both 3 and 6 mg/kg shortened reaction time by approximately 17–19 ms, with only an approximately 1.5-ms difference between doses, and both doses reduced RPE after the 60-m sprint. Systematic reviews provide relatively consistent evidence that caffeine improves attention, information-processing speed, and reaction speed in exercise settings [24,25], and meta-analytic and experimental evidence supports its ability to reduce perceived exertion [26,27]. The present findings indicate that the main benefits for response to the start signal and postexercise effort perception were already obtained at 3 mg/kg. In contrast, RFD continued to increase under 6 mg/kg, suggesting that central alertness, subjective perception, and rapid force generation after movement initiation differ in their dose sensitivity.

### Effects of caffeine across acceleration phases

The split-time findings form a coherent body of evidence with two recent sprint-specific studies. Matsumura et al. found that 6 mg/kg caffeine reduced adjusted 100-m time by 0.14 s, with most of the performance difference developing within the first 60 m and significant increases in mean velocity over 0–10 and 10–20 m [11]. Using step-by-step ground-reaction-force measurements, Horiuchi and Nagahara similarly found that 6 mg/kg caffeine improved performance throughout the 60-m acceleration phase; the changes included greater step frequency, shorter support and braking times, and greater propulsive force across several step intervals [16]. In the present study, 6 mg/kg reduced 60-m time by 0.175 s relative to placebo, and 3 mg/kg reduced it by 0.123 s. Relative to the placebo mean, these differences correspond to improvements of approximately 2.4% and 1.7%, respectively. For context, Tokutake and Ueta reported an intra-individual coefficient of variation of 1.40% for maximal 60-m sprint time across five testing days in collegiate male sprinters [32]. Although the protocols and timing systems differ, this provides a useful reference for interpreting the magnitude of the present group-mean changes alongside statistical significance. All 21 participants ran faster under CAF6, and 20 also improved under CAF3. The principal contribution beyond previous research is therefore that 3 mg/kg was sufficient to improve performance over the first 60 m in this cohort. The higher dose produced a greater cumulative benefit by 30 and 60 m, whereas the direct CAF6-versus-CAF3 contrast at 10 m was not significant in the primary crossover-adjusted model.

The earliest response occurred over 0–10 m, consistent with the components of this phase. Start timing includes the response to the start signal, the development of pressure against the blocks, block clearance, and the first steps of acceleration. Differences between world-class and well-trained sprinters are reflected not only in velocity after block clearance but also in block impulse, horizontal power, and the mechanical organization of the initial steps [28,29]. Both caffeine doses shortened start reaction time and increased starting-block RFD in the present study. These concurrent changes are consistent with earlier response initiation and more rapid horizontal force development accompanying the 0–10-m improvement, but they do not identify either variable as a mediator of the sprint-time effect. Because the direct CAF6-versus-CAF3 contrast for 10-m time was not significant in the primary mixed-effects model, the data do not support a distinct additional early-phase advantage of the higher dose despite its larger RFD response.

The 10–30-m interval remains part of the acceleration phase, but its mechanical demands shift from block clearance toward force orientation and support timing during successive steps. As velocity increases, ground-contact time decreases, and athletes must maintain effective propulsion within progressively shorter contact periods; the complete force-time waveform also changes systematically across acceleration phases within the same athlete [30,31]. Both CAF3 and CAF6 improved the 10–30-m split, but the doses did not differ significantly, suggesting that the improvements in initiation and rapid force generation produced by the lower dose covered much of the demand during this phase. Over 30–60 m, the primary mixed-effects model indicated improvements relative to placebo for both CAF3 and CAF6, although the CAF3 contrast was not retained in the robust GEE sensitivity analysis. The higher-dose effect was therefore more robust in this later interval. Neither caffeine dose differed significantly from the other for the 30–60-m split, indicating that the larger CAF6-versus-CAF3 difference in cumulative 60-m time was accumulated across preceding portions of the sprint rather than being attributable to a statistically distinct effect within the final split alone.

The absence of changes in mean step length and step frequency over the first 10 m does not contradict the step-frequency increases reported for selected step intervals by Horiuchi and Nagahara [16], because the studies differed in measurement resolution. Horiuchi and Nagahara analyzed support time, step frequency, and ground-reaction force in four-step intervals, whereas the present study obtained only mean step length and mean step frequency over the first 10 m. Averaging may obscure small adjustments in opposite directions among the initial steps and cannot characterize support time, flight time, braking force, or propulsive impulse. More importantly, the 10-m time in the present study included start reaction and the block phase, whereas step length and step frequency described running only after block clearance. Shorter sprint time in the absence of a change in mean spatiotemporal parameters is therefore not mathematically contradictory. A shorter reaction time, a faster block phase, or altered within-step contact structure could improve 10-m performance without a significant change in mean step length or frequency.

Accordingly, the present findings do not support attributing the sprint benefit simply to a longer step length or higher step frequency, but neither do they establish that kinematics were entirely unchanged. The available data identify concurrent changes in start reaction and rapid horizontal force generation, whereas step-by-step propulsive impulse, the ratio of force directed horizontally, contact time, and continuous velocity changes over 30–60 m were not measured. Sprint performance depends strongly on the direction and timing of force application, rather than only on maximal force or a single spatiotemporal average [5,6]. Future studies should synchronize starting-block force-time curves, step-by-step ground-reaction forces, and continuous velocity profiles to determine precisely which technical components are modified by 3 and 6 mg/kg caffeine.

### Relationships between neuromuscular outcomes and sprint performance

Individual improvements in 60-m performance were not significantly correlated with changes in CMJ, SJ, Wingate power, start reaction time, or any RFD measure. This finding does not exclude the involvement of these outcomes in caffeine-mediated performance enhancement, but it argues against an overly simple explanation: the present data do not show that athletes with a larger improvement in any single outcome necessarily achieved a greater 60-m benefit. Group-level treatment effects and correlations among individual change scores address different questions. Caffeine may improve the mean of several outcomes simultaneously, while the route through which individual athletes achieve a performance benefit may differ.

From a measurement perspective, peak RFD represents only the steepest portion of the force-time curve. It is not equivalent to total block impulse and does not indicate whether force was directed effectively in the horizontal direction. RFD is sensitive to the detection of force onset, filtering, analysis window, and trial selection, and rapid-force measures generally show greater relative error than peak force or peak power [14,22]. In contrast, 60-m performance integrates reaction, the block phase, continuous acceleration, and running at higher velocity. Using a single slope-related starting-block variable to explain the entire 60-m response therefore creates a mismatch in measurement level. The null correlations suggest that future mechanistic analyses should prioritize the combined changes in horizontal impulse, force orientation, step-by-step contact time, and velocity profile rather than treating RFD as the sole mediator.

The vertical jump findings require the same boundary of interpretation. CMJ and SJ height both increased significantly, but jump height does not establish that every power-related variable improved [17,20]. Force-platform waveforms from the jumps were not retained, precluding determination of whether caffeine altered countermovement depth, push-off duration, or peak velocity. The plateau in Wingate mean power further indicates that ‘power’ across different tasks does not represent a single physiological construct. The interpretation most consistent with the present data is that caffeine improved several forms of rapid performance, but that these outcomes were not linked through a single proportional transfer pathway.

RPE was a subjective outcome and may also have been influenced by blinding and expectancy. Although visually identical capsules were used, participants were not asked to identify the condition received. In Grgic’s CMJ study, a relatively high proportion of participants correctly identified caffeine or placebo after testing, whereas Muñoz et al. achieved more successful blinding in handball players [17,18]. Thus, even in a double-blind design, the success of blinding may vary with dose, participant experience, and physiological sensations. At 6 mg/kg, more noticeable sensations could have increased the chance of partial unblinding in athletes familiar with caffeine use. Placebo and expectancy effects can account for part of the overall response to ergogenic interventions in caffeine research [33,34]. The reduction in RPE should therefore be interpreted as a subjective treatment response that may include both pharmacological and expectancy-related components. Objective outcomes including 10–60-m times and RFD improved concurrently, indicating that the main conclusions did not depend on subjective reporting.

### Limitations

This study has several limitations. First, testing ended at 60 m; therefore, the effects of caffeine on maximal-velocity maintenance and deceleration over 60–100 m could not be determined. In addition, the 0–10-, 10–30-, and 30–60-m splits were obtained by subtracting cumulative times and are subject to greater error than directly measured independent splits. Second, the 60-m sprint, CMJ, SJ, and Wingate test were completed in a fixed order. Because no within-session pre-sprint CMJ, SJ, or Wingate baseline was collected, the fatigue decrement induced by the preceding sprint cannot be quantified, and these secondary outcomes should be interpreted as responses within a standardized post-sprint sequence rather than as independent rested-state measures. Third, plasma caffeine was not measured, habitual caffeine intake was recorded but was not examined as a moderator of the ergogenic response, and the success of blinding and adverse effects were not systematically assessed. Cohort-level numerical starting-block distances and angles were unavailable for quantitative analysis, although each participant used the same individualized block setup across conditions. Meta-analytic evidence generally indicates that habitual caffeine intake does not meaningfully attenuate the acute ergogenic effect, although individual responses may still be influenced by metabolic, genetic, and expectancy-related factors [35,36]. Higher caffeine doses also increase the incidence of adverse effects such as insomnia, nervousness, and palpitations, and caffeine ingested later in the day may substantially impair subsequent sleep [37,38]. Because adverse effects and tolerability were not systematically assessed, the present study characterizes performance responses rather than the relative benefit-risk or tolerability profile of the two doses. Scheduling trials at similar times of day within participants reduced between-condition time-of-day variation, but exact clock times, chronotype, habitual sleep-wake timing, recent sleep duration, and time since waking were not recorded. The sessions were therefore not standardized to biological phase. Recent studies show that time of day can influence neuromuscular performance and that chronobiological standardization can affect interpretation of acute caffeine responses [39,40]. Future work should consider chronotype or objective circadian phenotyping and scheduling relative to habitual wake time.

Fourth, the three-sequence cyclic crossover allocation balanced treatment across periods but did not include all six possible treatment sequences. As a result, a full first-order carryover effect could not be estimated independently from the direct treatment effect. The washout interval of at least 7 days, the absence of significant period or sequence effects for continuous outcomes, and the directionally concordant first-period sensitivity analysis reduce concern about material carryover but cannot prove its absence. Fifth, pre-sprint baseline RPE was not collected. The significant period effect in the ordinal RPE model therefore indicates that visit-related changes in perceived exertion may partly reflect unmeasured differences in baseline fatigue or readiness, even though the treatment effect remained significant after period adjustment. Sixth, the sample size was feasibility-based and no formal a priori power calculation was performed; although the crossover design increases within-participant efficiency, the modest sample limits precision for small treatment differences, particularly comparisons between the two active caffeine doses.

## Conclusions

Acute ingestion of 3 and 6 mg/kg caffeine improved performance over the first 60 m, vertical jump height, 10-s Wingate power, start reaction time, and starting-block RFD in trained male 100-m sprinters, while reducing perceived exertion after the 60-m sprint. Compared with 3 mg/kg, 6 mg/kg produced greater improvements in cumulative 30- and 60-m times, vertical jump performance, and RFD, whereas the two doses did not differ significantly for the 10–30- or 30–60-m split times. The higher dose did not provide additional benefits for Wingate mean power, start reaction time, or RPE, and neither dose significantly altered mean step length or step frequency over the first 10 m. Thus, the dose response was outcome-specific rather than uniformly greater at 6 mg/kg. A dose of 3 mg/kg was sufficient to improve multiple performance outcomes in this cohort, while 6 mg/kg provided additional benefits for selected outcomes. These sprint findings are limited to the first 60 m, and the study did not compare the tolerability profiles of the two doses.

## Supporting information

S1 Checklist CONSORT 2025 checklist for the randomized crossover trial. (DOCX)

S2 Checklist TIDieR checklist describing the caffeine and placebo interventions. (DOCX)

S1 Protocol English-language study protocol corresponding to the study procedures approved by the Sports Science Experimental Ethics Committee of Beijing Sport University. (PDF)

S1 Table Exploratory correlations between placebo-referenced improvement in 60-m sprint time and changes in candidate performance outcomes. (DOCX)

S1 Data De-identified participant-period data, including randomized sequence and period variables used in the crossover analyses, together with a data dictionary and aggregate baseline summary. (ZIP)

S1 Code Python code used to reproduce the crossover-adjusted mixed-effects analyses, robustness analyses, ordinal RPE model, carryover identifiability check, first-period sensitivity summary, and exploratory correlation table. (ZIP)
