# Supplementary material for: Acute doses of 3 and 6 mg/kg caffeine produce outcome- and phase-specific improvements in male 100-m sprinters: A randomized, double-blind, placebo-controlled crossover trial
Source: PLoS One. 2026 Nov 10;21(11):e0361864. doi: 10.1371/journal.pone.0361864 (PMC13750558; doi:10.1371/journal.pone.0361864)
Supplement: Supplementary file 1 [file pone.0361864.s003.pdf]

STUDY PROTOCOL

Acute Effects of 3 and 6 mg/kg Caffeine on Sprint and Lower-Limb Explosive Performance in Male 100-m Sprinters

A randomized, double-blind, placebo-controlled, three-condition crossover trial

**Protocol version**	Version 1.0
**Ethics approval**	Sports Science Experimental Ethics Committee of Beijing Sport University; approval no. 2026355H; approval date: 1 May 2026
**Trial registration**	Chinese Clinical Trial Registry; ChiCTR2600128594; registered 22 July 2026; https://www.chictr.org.cn/showproj.html?proj=333474
**Study institution**	Beijing Sport University, Beijing, China
**Investigators**	Tao Mei; Jianing Li; Xiangang Yang
**Target population**	Male 100-m sprinters meeting the Chinese national second-class athlete standard
**Planned sample**	21 participants

Protocol provenance statement. This English-language protocol document was prepared for journal submission from the study procedures and original statistical analysis plan approved by the Sports Science Experimental Ethics Committee of Beijing Sport University under approval no. 2026355H. It is not the committee-issued ethics approval document. The original approved analysis framework is reproduced below. A crossover-adjusted statistical reanalysis was introduced after trial completion during peer review and is disclosed in the main manuscript and S5 Code; it is not represented here as part of the original analysis plan. The committee-issued ethics approval document was provided separately to the journal.

Supporting Information file: S2 Protocol

1. Protocol summary

Protocol element	Description
**Study design**	Randomized, double-blind, placebo-controlled, three-condition crossover trial using three balanced cyclic treatment sequences (A, B, C)
**Conditions**	Placebo (PLA; 0 mg/kg caffeine), 3 mg/kg caffeine (CAF3), and 6 mg/kg caffeine (CAF6)
**Visits**	One familiarization visit and three experimental trials
**Washout**	At least 7 days between experimental trials
**Timing of supplementation**	Capsules ingested with 200 mL water; standardized warm-up began 30 min after ingestion; the 60-m sprint test began 60 min after ingestion
**Primary performance outcomes**	Cumulative 10-, 30-, and 60-m sprint times
**Secondary outcomes**	Start reaction time; peak horizontal, front-block peak, and rear-block peak rate of force development; first-10-m mean step length and step frequency; post-sprint rating of perceived exertion; countermovement jump and squat jump height; 10-s Wingate peak and mean power
**Exploratory outcomes**	Derived 0-10-, 10-30-, and 30-60-m split times; placebo-referenced change-score correlations
**Analysis population**	All randomized participants completing the three conditions with available paired observations

2. Background and rationale

Caffeine is a widely used acute ergogenic aid in competitive sport. Its principal mechanism is antagonism of adenosine receptors, which may increase alertness and neural activation and reduce pain and perceived exertion. Doses of 3-6 mg/kg body mass are commonly recommended approximately 60 min before exercise, but the ergogenic response is not necessarily linear across this range and may depend on the task and performance outcome.

The 100-m sprint consists of distinct phases, including response to the start signal, block clearance, initial acceleration, attainment of maximal velocity, and velocity maintenance. Performance during the first 60 m depends strongly on the rapid production and orientation of force within progressively shorter support times. Therefore, a caffeine-induced improvement in final sprint time may originate from different phases and may not be accompanied by uniform changes in reaction time, starting-block force development, step characteristics, jumping performance, cycling power, or perceived exertion.

Most prior caffeine studies have examined repeated-sprint protocols or athletes from intermittent team sports. Evidence from trained track sprinters and direct comparisons of 3 and 6 mg/kg caffeine remains limited. The present protocol was designed to compare both doses under standardized field-laboratory conditions and to determine whether any additional benefit of 6 mg/kg is outcome-specific or phase-specific.

3. Objectives

3.1 Primary objective

To compare the acute effects of placebo, 3 mg/kg caffeine, and 6 mg/kg caffeine on cumulative 10-, 30-, and 60-m sprint times in trained male 100-m sprinters.

3.2 Secondary objectives

- To compare the three conditions for start reaction time and starting-block rate-of-force-development measures.

- To compare the three conditions for first-10-m mean step length and step frequency.
- To compare the three conditions for countermovement jump height, squat jump height, and 10-s Wingate peak and mean power.
- To compare post-sprint rating of perceived exertion among the three conditions.

3.3 Exploratory objectives

- To characterize caffeine effects across the derived 0-10-, 10-30-, and 30-60-m sprint segments.
- To examine whether placebo-referenced improvements in 60-m performance are associated with corresponding changes in neuromuscular and performance outcomes.

4. Study design and setting

This was a randomized, double-blind, placebo-controlled, three-condition crossover trial conducted at Beijing Sport University. Each participant completed one familiarization visit followed by three experimental trials. Experimental trials were scheduled at similar times of day for each participant and were separated by at least 7 days to minimize residual effects from the preceding supplementation and testing session. Experimental controls, supplementation timing, testing procedures, and the order of outcome assessments were kept identical across conditions.

The study used a within-participant design in which every participant completed PLA, CAF3, and CAF6. Allocation was implemented in blocks of three using three cyclic treatment sequences: A (PLA → CAF3 → CAF6), B (CAF3 → CAF6 → PLA), and C (CAF6 → PLA → CAF3), with seven participants assigned to each sequence. This ensured that each treatment appeared equally often in each experimental period.

5. Participants

5.1 Eligibility criteria

Inclusion criteria were:

1. Male, 18-25 years of age.
2. Performance meeting the Chinese national second-class standard for the 100 m during the study period.
3. At least 3 years of systematic sprint training.
4. Willingness to participate and ability to complete all testing procedures.

Exclusion criteria were:

5. Known caffeine allergy or a history of marked adverse responses to caffeine ingestion.
6. An open or closed lower-limb joint injury within the previous 3 months.
7. Illness, pain, or other discomfort before testing that could impair maximal exercise.

5.2 Recruitment, consent, and withdrawal

Potential participants were informed verbally and in writing about the study purpose, procedures, potential benefits, and foreseeable risks. Written informed consent was required before enrollment. Participation was voluntary, and participants could withdraw at any time without penalty. A testing session could be stopped by the investigator if a participant developed pain, illness, marked discomfort, or any condition making maximal exercise unsafe.

5.3 Planned sample

The protocol planned to enroll 21 eligible male 100-m sprinters and to obtain complete observations under all three experimental conditions.

6. Allocation and blinding

6.1 Allocation

Participants were allocated by drawing lots in blocks of three to one of three cyclic sequences: A (PLA → CAF3 → CAF6), B (CAF3 → CAF6 → PLA), or C (CAF6 → PLA → CAF3), with seven participants in each sequence. A washout period of at least 7 days separated trials.

6.2 Blinding

Caffeine and placebo capsules were weighed, prepared, and coded by a researcher who was not directly involved in on-site testing. Participants and staff responsible for capsule administration, performance testing, and data recording were unaware of capsule content. Caffeine and placebo were supplied in visually identical white capsules.

7. Interventions

Condition	Dose	Capsule content
**PLA**	0 mg/kg caffeine	White flour enclosed in identical white capsules
**CAF3**	3 mg/kg body mass	Commercially available caffeine capsules prepared according to body mass
**CAF6**	6 mg/kg body mass	Commercially available caffeine capsules prepared according to body mass

On each experimental day, participants rested quietly for 15 min and then swallowed the assigned capsules with 200 mL water. A standardized warm-up began 30 min after ingestion. The all-out 60-m sprint test began 60 min after ingestion.

8. Pretrial standardization and compliance

- Trials for each participant were scheduled at similar times of day.
- Participants maintained their usual training program but refrained from strenuous exercise and alcohol for 24 h before each trial.
- Participants avoided sport-nutrition supplements other than those specified in the protocol.
- For 48 h before each trial, participants abstained from coffee, tea, energy drinks, cola, chocolate, and other caffeine-containing foods or beverages.
- Participants refrained from eating for at least 2 h before arrival.
- Foods and beverages consumed before the first trial were recorded, and participants were instructed to replicate the same foods and meal timing before subsequent trials as closely as possible.
- Before each trial, investigators verified recent health status, caffeine intake, alcohol consumption, strenuous exercise, and supplement use verbally.
- Indoor temperature and relative humidity were recorded, and testing equipment was inspected before each trial.

9. Visit schedule and experimental procedures

Visit/time	Procedure
**Familiarization visit**	Explanation and practice of all tests; adjustment and recording of starting-block and ergometer settings; standardized instructions.
**Experimental trial -15 min**	Arrival and 15-min seated rest; compliance and health-status checks.
**0 min**	Ingestion of assigned capsules with 200 mL water.

**+30 min**	Standardized warm-up consisting primarily of low-intensity jogging and dynamic stretching sufficient to induce light perspiration without fatigue.
**+60 min**	Two maximal 60-m sprint trials separated by 5 min; cumulative timing, reaction time, starting-block force variables, first-10-m step variables, and RPE collected.
**Post-sprint +10 min**	Countermovement jump, squat jump, and 10-s Wingate test completed in fixed order with 3 min rest between tests.

10. Outcome measures

10.1 Sprint times and split times

Sprint testing was conducted on an indoor track. Participants used a crouch start from Kistler starting blocks with the corresponding starting system. A SmartSpeed portable timing system recorded cumulative times at 10, 30, and 60 m. Participants completed two valid maximal 60-m trials separated by 5 min, and the trial with the faster 60-m time was retained for analysis. Derived split times were calculated as follows: 0-10 m = cumulative 10-m time; 10-30 m = cumulative 30-m time minus cumulative 10-m time; 30-60 m = cumulative 60-m time minus cumulative 30-m time.

10.2 Start reaction time and rate of force development

Start reaction time and starting-block force variables were acquired using the Kistler sprint-start system and processed in KiSprint software (version 4.1.0.12). Start reaction time was defined as the interval between presentation of the start stimulus and the system-detected onset of movement. The system automatically calculated rate of force development from the force-time data. Peak horizontal RFD, front-block peak RFD, and rear-block peak RFD were retained.

10.3 Step length and step frequency

Kinematics during the first 10 m were measured using an OptoJump optical measurement system synchronized with the starting system. Step length was the horizontal distance between the toe of the support foot and the toe of the contralateral foot at the subsequent ground contact. Step frequency was calculated as 1/(ground-contact time + flight time). A representative mean step length and step frequency for the first 10 m were retained under each condition.

10.4 Rating of perceived exertion

Overall perceived exertion was assessed immediately after each 60-m sprint using the Borg 6-20 RPE scale after standardized explanation of the scale anchors.

10.5 Vertical jump performance

Countermovement jump and squat jump were performed on a Kistler portable three-dimensional force platform. Three valid trials were completed for each jump, and the greatest jump height calculated by the force-platform software was retained. Trials with clear technical errors were repeated.

10.6 10-s Wingate power

Anaerobic power was assessed using a 10-s Wingate test on a Monark 894E cycle ergometer. The load factor was 0.090 kg per kilogram of body mass. Peak power and mean power were recorded in watts. Saddle position was individualized during familiarization and reproduced at all trials.

11. Safety monitoring

Eligibility screening excluded individuals with known caffeine allergy, marked previous adverse responses, recent lower-limb injury, or current illness or discomfort that could compromise maximal

exercise. Health status and protocol compliance were checked before every trial. Testing was to be stopped if a participant developed pain, marked discomfort, or any sign that maximal exercise should not continue. A formal structured adverse-event questionnaire was not planned; spontaneously reported symptoms and any event requiring test modification or termination were to be documented.

12. Statistical analysis plan

Continuous variables were to be summarized as mean ± standard deviation. RPE was to be summarized as median with the 25th and 75th percentiles. Analyses were two-sided with statistical significance set at $\alpha = 0.05$.

- Continuous outcomes: one-way repeated-measures analysis of variance with condition (PLA, CAF3, CAF6) as the within-participant factor.
- Assumptions: residual histograms, Q-Q plots, and the Shapiro-Wilk test for residual normality; Mauchly test for sphericity; Greenhouse-Geisser correction when sphericity was violated.
- Omnibus effects: F value, corrected degrees of freedom, p value, and partial η^2.
- Pairwise comparisons: paired t tests with Holm adjustment; mean difference, 95% confidence interval, and paired Hedges g_z.
- Confidence intervals for Hedges g_z: participant-level bootstrap resampling.
- RPE: Friedman test with Kendall W; pairwise Wilcoxon signed-rank tests with Holm adjustment.
- Multiplicity: Benjamini-Hochberg false-discovery-rate adjustment for omnibus p values of the main outcomes and for exploratory correlation analyses.
- Split times: analyzed as exploratory outcomes using repeated-measures analysis of variance and Holm-adjusted pairwise comparisons.
- Sensitivity analyses: Friedman and Wilcoxon signed-rank tests for continuous outcomes.
- Exploratory associations: Spearman rank correlations between placebo-referenced improvement in 60-m time and corresponding changes in jump height, Wingate power, start reaction time, and RFD outcomes.

The planned analysis population comprised all randomized participants completing all three conditions with available paired observations. No interim analyses or formal stopping guidelines were planned. Missing observations, if any, were to be reported; no imputation was planned for the primary within-participant analyses. Analyses were to be conducted in Python using pandas, SciPy, and statsmodels.

13. Data management and confidentiality

Each participant was assigned a study identifier. Analytical datasets were to use study identifiers rather than directly identifying information. Consent forms and identifying information were to be stored separately from performance data. Electronic data were to be maintained on password-protected institutional or investigator-controlled storage with access limited to the research team. Data checks were to include verification of participant-condition matching, range checks, and review of unusual values against source records when available.

De-identified data and analysis code may be shared for scientific verification subject to institutional ethical requirements, participant privacy protections, and journal policy.

14. Ethical considerations

The study was conducted in accordance with the Declaration of Helsinki and applicable institutional requirements. Approval was obtained from the Sports Science Experimental Ethics Committee of Beijing Sport University (approval no. 2026355H; approval date: 1 May 2026). Written informed consent was required from all participants before enrollment. The trial was subsequently registered in the Chinese Clinical Trial Registry on 22 July 2026 (ChiCTR2600128594).

15. Dissemination

Results were intended for dissemination through a peer-reviewed scientific journal and academic presentations. Reports were to present aggregate results and de-identified individual-level data where appropriate. Authorship was to reflect substantive contributions to study conception, methodology, investigation, analysis, interpretation, and manuscript preparation.

16. Protocol amendments and deviations

No substantive amendments were made during trial conduct to the intervention conditions, test sequence, outcome definitions, or randomization scheme. After trial completion, during peer review, the primary statistical analysis was amended from the protocol-specified one-way repeated-measures ANOVA to a crossover-adjusted linear mixed-effects framework so that treatment period and randomized sequence were modeled explicitly. This post-study statistical amendment is disclosed in the main manuscript and S5 Code and does not alter the original analysis plan reproduced in Section 12.

17. Key references

1. Guest NS, VanDusseldorp TA, Nelson MT, et al. International Society of Sports Nutrition position stand: caffeine and exercise performance. J Int Soc Sports Nutr. 2021;18:1.
2. Graham TE, Spriet LL. Metabolic, catecholamine, and exercise performance responses to various doses of caffeine. J Appl Physiol. 1995;78:867-874.
3. Matsumura T, Tomoo K, Sugimoto T, et al. Acute effect of caffeine supplementation on 100-m sprint running performance: a field test. Med Sci Sports Exerc. 2023;55:525-533.
4. Horiuchi M, Nagahara R. Acute effects of caffeine supplementation on kinematics and kinetics of sprinting. Scand J Med Sci Sports. 2024;34:e14595.
5. Maffiuletti NA, Aagaard P, Blazevich AJ, et al. Rate of force development: physiological and methodological considerations. Eur J Appl Physiol. 2016;116:1091-1116.
6. Pallarés JG, Fernández-Elías VE, Ortega JF, et al. Neuromuscular responses to incremental caffeine doses: performance and side effects. Med Sci Sports Exerc. 2013;45:2184-2192.

Protocol confirmation

The undersigned authors confirm that this English-language document accurately presents the study procedures and original statistical analysis plan approved by the Sports Science Experimental Ethics Committee of Beijing Sport University under approval no. 2026355H. The post-study statistical amendment introduced during peer review is clearly identified in Section 16 and is reported in the main manuscript and S5 Code.

Investigator	Signature	Date
Tao Mei		
Jianing Li		
Xiangang Yang		
