# Supplementary material for: Acute doses of 3 and 6 mg/kg caffeine produce outcome- and phase-specific improvements in male 100-m sprinters: A randomized, double-blind, placebo-controlled crossover trial
Source: PLoS One. 2026 Nov 10;21(11):e0361864. doi: 10.1371/journal.pone.0361864 (PMC13750558; doi:10.1371/journal.pone.0361864)
Supplement: Supplementary file 2 [file pone.0361864.s001.docx]

**S1 Checklist. CONSORT 2025 checklist**

*Acute doses of 3 and 6 mg/kg caffeine produce outcome- and phase-specific improvements in male 100-m sprinters: a randomized, double-blind, placebo-controlled crossover trial*

Page and continuous line numbers refer to PLOS_ONE_Manuscript_Revised_Clean.docx. Items not applicable or not reported are marked N/A. The checklist is used together with the CONSORT extension for randomized crossover trials.

| **Section/topic** | **No.** | **CONSORT 2025 item description** | **Reported on Page Number/Line Number** | **Reported on Section/Paragraph** |
| --- | --- | --- | --- | --- |
| Title and structured abstract | 1a | Identification as a randomized trial | Page 1, lines 1-3 | Title |
| Title and structured abstract | 1b | Structured summary of trial design, methods, results, and conclusions | Page 2, lines 14-35 | Abstract |
| Trial registration | 2 | Name of trial registry, identifying number, and date of registration | Page 2, lines 34-35; pages 7-8, lines 161-175 | Abstract; Materials and Methods - Participants |
| Protocol and statistical analysis plan | 3 | Where the trial protocol and statistical analysis plan can be accessed | Page 30, lines 750-760 | Supporting information captions; S2 Protocol; S5 Code |
| Data sharing | 4 | Where and how de-identified participant data, data dictionary, statistical code, and other materials can be accessed | Page 30, lines 756-760 | Supporting information captions; S4 Data and S5 Code |
| Funding and conflicts of interest | 5a | Sources of funding and other support and the role of funders | N/A | PLOS submission system - Financial Disclosure |
| Funding and conflicts of interest | 5b | Financial and other conflicts of interest of the manuscript authors | N/A | PLOS submission system - Competing Interests |
| Background and rationale | 6 | Scientific background and rationale | Pages 2-5, lines 36-108 | Introduction |
| Objectives | 7 | Specific objectives related to benefits and harms | Page 5, lines 109-118 | Introduction, final paragraph |
| Patient and public involvement | 8 | Details of patient or public involvement in design, conduct, and reporting | N/A | N/A |
| Trial design | 9 | Description of trial design, allocation, and framework | Page 6, lines 121-142 | Materials and Methods - Study Design |
| Changes to trial protocol | 10 | Important changes to the trial after commencement, including outcomes or analyses not prespecified, with reasons | S2 Protocol (original analysis plan); pages 11-12, lines 258-289 (final analysis); Response to Reviewers (reason for change) | Statistical analysis change documented across protocol, final Methods, and revision response |
| Trial setting | 11 | Settings and locations where the trial was conducted | Pages 7-11, lines 143-257 | Participants; Experimental Procedures; Outcome Measures |
| Eligibility criteria | 12a | Eligibility criteria for participants | Page 7, lines 144-151 | Participants, paragraph 1 |
| Eligibility criteria | 12b | Eligibility criteria for sites and for individuals delivering the interventions, if applicable | N/A | N/A |
| Intervention and comparator | 13 | Intervention and comparator details sufficient for replication | Pages 6-9, lines 121-204 | Study Design; Pretrial Controls; Experimental Procedures; Fig 1B |
| Outcomes | 14 | Prespecified primary and secondary outcomes, measurement variables, analysis metrics, aggregation methods, and time points | Pages 9-12, lines 206-289 | Outcome Measures; Statistical Analysis |
| Harms | 15 | How harms were defined and assessed | Page 13, line 302; page 25, lines 617-628 | Results, opening paragraph; Limitations - adverse events not systematically assessed |
| Sample size | 16a | How sample size was determined, including assumptions supporting any calculation | Page 7, lines 152-160 | Participants - sample-size rationale and sensitivity benchmark |
| Sample size | 16b | Interim analyses and stopping guidelines | N/A | N/A |
| Sequence generation | 17a | Who generated the random allocation sequence and the method used | Page 6, lines 124-127 | Study Design - allocation by lot in blocks of three |
| Sequence generation | 17b | Type of randomization and details of restrictions | Page 6, lines 124-127 | Study Design - three cyclic sequences, seven participants per sequence |
| Allocation concealment mechanism | 18 | Mechanism used to implement and conceal the random allocation sequence until assignment | Page 6, lines 124-127 | Study Design - allocation by lot; concealment mechanism not separately documented |
| Implementation | 19 | Whether enrollment and assignment personnel had access to the random allocation sequence | Not separately reported | Enrollment/assignment access to the sequence was not separately documented |
| Blinding | 20a | Who was blinded after assignment to interventions | Page 6, lines 132-134 | Study Design |
| Blinding | 20b | How blinding was achieved and similarity of interventions | Page 9, lines 193-198 | Experimental Procedures |
| Statistical methods | 21a | Statistical methods used for primary and secondary outcomes, including harms | Pages 11-12, lines 258-289 | Statistical Analysis |
| Statistical methods | 21b | Definition of who was included in each analysis and in which group | Page 12, lines 286-288; page 13, lines 297-300 | Statistical Analysis; Results, opening paragraph |
| Statistical methods | 21c | How missing data were handled | Page 12, lines 287-288; page 13, lines 297-300 | Statistical Analysis; Results, opening paragraph |
| Statistical methods | 21d | Methods for additional analyses, distinguishing prespecified from post hoc | Pages 11-12, lines 269-289 | Statistical Analysis - robustness, exploratory, and sensitivity analyses |
| Participant flow | 22a | For each group/condition, numbers randomly assigned, receiving intended intervention, and analyzed | Page 6, lines 136-140; page 13, lines 297-300 | Fig 1A caption; Results, opening paragraph |
| Participant flow | 22b | Losses and exclusions after randomization, with reasons | Page 6, lines 136-140; page 13, lines 297-300 | Fig 1A caption; Results, opening paragraph |
| Recruitment | 23a | Dates defining recruitment and follow-up periods | Pages 7-8, lines 161-175 | Participants - recruitment period and trial registration |
| Recruitment | 23b | Why the trial ended or was stopped, if relevant | N/A | N/A |
| Intervention and comparator delivery | 24a | Intervention and comparator as actually administered, including adherence and fidelity | Pages 8-9, lines 177-204; page 13, lines 297-300 | Pretrial Controls; Experimental Procedures; Results, opening paragraph |
| Intervention and comparator delivery | 24b | Concomitant care received during the trial for each condition | Page 8, lines 177-191 | Pretrial Controls |
| Baseline data | 25 | Baseline demographic and clinical characteristics | Page 8, line 176 | Table 1 |
| Numbers analyzed, outcomes, and estimation | 26 | For each outcome: number analyzed, available data, result by group/condition, effect estimate, and precision | Pages 13-17, lines 311-422; Figs 2-4 | Results; Figs 2-4; S3 Table |
| Harms | 27 | All harms or unintended events in each group/condition | Page 13, line 302 | Results, opening paragraph |
| Ancillary analyses | 28 | Other analyses, including subgroup and sensitivity analyses, distinguishing prespecified from post hoc | Page 13, lines 304-308; pages 14-15, lines 342-361; page 17, lines 416-422 | First-period sensitivity analysis; exploratory split-time and correlation analyses |
| Interpretation | 29 | Interpretation consistent with results, balancing benefits and harms and considering other evidence | Pages 17-24, lines 423-607 | Discussion |
| Limitations | 30 | Trial limitations, including potential bias, imprecision, generalizability, and multiplicity | Pages 24-25, lines 608-640 | Limitations |
