# Supplementary material for: Acute doses of 3 and 6 mg/kg caffeine produce outcome- and phase-specific improvements in male 100-m sprinters: A randomized, double-blind, placebo-controlled crossover trial
Source: PLoS One. 2026 Nov 10;21(11):e0361864. doi: 10.1371/journal.pone.0361864 (PMC13750558; doi:10.1371/journal.pone.0361864)
Supplement: Supplementary file 3 [file pone.0361864.s002.docx]

**S6 Checklist. TIDieR checklist**

Template for Intervention Description and Replication checklist for the caffeine and placebo interventions.

| **Item** | **TIDieR item** | **Where reported** | **Details** |
| --- | --- | --- | --- |
| 1 | Brief name | Methods - Study Design and Experimental Procedures | Placebo, 3 mg/kg caffeine, and 6 mg/kg caffeine conditions. |
| 2 | Why | Introduction | Adenosine-receptor antagonism and uncertainty regarding outcome- and phase-specific caffeine dose responses. |
| 3 | Materials | Methods - Experimental Procedures | Nutricost caffeine; white flour placebo; identical white capsules; 200 mL water. |
| 4 | Procedures | Methods - Pretrial Controls, Experimental Procedures, and Outcome Measures; Fig 1B | Pretrial controls, capsule ingestion, warm-up, sprint, recovery, jump, and Wingate procedures. |
| 5 | Provider | Methods - Study Design and Experimental Procedures | Capsules prepared and coded by a researcher not involved in on-site testing; capsule administration and testing personnel were blinded. |
| 6 | Mode of delivery | Methods - Experimental Procedures | Oral capsules swallowed with 200 mL water. |
| 7 | Location | Methods - Participants and Outcome Measures | Beijing Sport University indoor track and laboratory testing facilities. |
| 8 | When and how much | Methods - Study Design and Experimental Procedures | Single acute dose; testing 60 min after ingestion; three experimental trials separated by at least 7 days. |
| 9 | Tailoring | Methods - Experimental Procedures and Outcome Measures | Caffeine dose individualized to body mass; starting-block and cycle-ergometer settings individualized and reproduced. |
| 10 | Modifications | N/A | No intervention modifications were reported. |
| 11 | Adherence | Methods - Pretrial Controls and Experimental Procedures | Pretrial compliance was verified verbally; capsule ingestion was supervised. |
| 12 | Actual delivery | Results, opening paragraph; Fig 1A | All 21 participants completed all three conditions; there were no post-randomization exclusions. |
