# Supplementary material for: Acute doses of 3 and 6 mg/kg caffeine produce outcome- and phase-specific improvements in male 100-m sprinters: A randomized, double-blind, placebo-controlled crossover trial
Source: PLoS One. 2026 Nov 10;21(11):e0361864. doi: 10.1371/journal.pone.0361864 (PMC13750558; doi:10.1371/journal.pone.0361864)
Supplement: Supplementary file 4 [file pone.0361864.s004.docx]

Supplementary Table S1

Exploratory correlations between improvement in 60-m sprint time and changes in candidate performance outcomes

| **Caffeine condition** | **Candidate outcome** | **n** | **Spearman ρ** | **Raw p** | **BH-FDR q** | **FDR significant** |
| --- | --- | --- | --- | --- | --- | --- |
| CAF3 | CMJ height | 21 | 0.343 | 0.128 | 0.577 | No |
| CAF3 | SJ height | 21 | 0.000 | 1.000 | 1.000 | No |
| CAF3 | Wingate peak power | 21 | -0.131 | 0.571 | 0.894 | No |
| CAF3 | Wingate mean power | 21 | -0.149 | 0.518 | 0.894 | No |
| CAF3 | Start reaction time | 21 | 0.035 | 0.880 | 0.990 | No |
| CAF3 | Horizontal maximum RFD | 21 | -0.109 | 0.638 | 0.894 | No |
| CAF3 | Horizontal mean RFD | 21 | -0.145 | 0.529 | 0.894 | No |
| CAF3 | Front-block maximum RFD | 21 | -0.091 | 0.695 | 0.894 | No |
| CAF3 | Rear-block maximum RFD | 21 | 0.413 | 0.063 | 0.565 | No |
| CAF6 | CMJ height | 21 | 0.057 | 0.808 | 0.808 | No |
| CAF6 | SJ height | 21 | 0.095 | 0.683 | 0.808 | No |
| CAF6 | Wingate peak power | 21 | -0.168 | 0.468 | 0.808 | No |
| CAF6 | Wingate mean power | 21 | -0.405 | 0.069 | 0.619 | No |
| CAF6 | Start reaction time | 21 | 0.062 | 0.788 | 0.808 | No |
| CAF6 | Horizontal maximum RFD | 21 | -0.188 | 0.414 | 0.808 | No |
| CAF6 | Horizontal mean RFD | 21 | 0.190 | 0.410 | 0.808 | No |
| CAF6 | Front-block maximum RFD | 21 | -0.287 | 0.207 | 0.808 | No |
| CAF6 | Rear-block maximum RFD | 21 | 0.085 | 0.714 | 0.808 | No |

*Note. 60-m improvement = PLA time − caffeine-condition time (positive = faster). For CMJ, SJ, Wingate power, and RFD, change = caffeine − PLA; for reaction time, change = PLA − caffeine so that positive values consistently indicate improvement.*

*BH-FDR correction was applied separately to the nine correlations within each caffeine dose. No association remained significant after FDR correction.*

*Abbreviations: BH-FDR, Benjamini–Hochberg false discovery rate; CAF3, 3 mg/kg caffeine; CAF6, 6 mg/kg caffeine; CMJ, countermovement jump; PLA, placebo; RFD, rate of force development; SJ, squat jump.*

*n = 21 complete paired observations per correlation.*
